# Characterisation of Polishing Frequency for Three Varieties of Sorghum Grain in Java, Indonesia

**DOI:** 10.1155/2022/2949665

**Published:** 2022-06-27

**Authors:** Nur Arifah Qurota A'yunin, Taufiq Firdaus Al-Ghifari Atmadja, Nur Aini, Pepita Haryanti

**Affiliations:** ^1^Department of Agrotechnology, Siliwangi University, Tasikmalaya, West Java 46196, Indonesia; ^2^Department of Nutrition, Siliwangi University, Tasikmalaya, West Java 46196, Indonesia; ^3^Department of Food Technology, Jenderal Soedirman University, Purwokerto, Central Java 53123, Indonesia

## Abstract

To determine the suitability of different sorghum cultivars (grown in Indonesia) for the manufacturing of acceptable food products, their properties must first be characterised. During sorghum processing, polishing may affect the final nutritional value and quality of the product. This study is aimed at determining the effects of sorghum variety and polishing frequency on nutritional value. This was achieved by using a factorial randomised block design with two factors: sorghum variety (Bioguma, Numbu, and Super) and polishing frequency (once, twice, or thrice). Tannin content, antioxidant capacity, levels of dietary fibre and resistant starch, and fat, ash, and carbohydrate content varied according to sorghum cultivar. Compared to other sorghum varieties, the Super cultivar contained the highest levels of antioxidants, dietary fibre, fat content, protein, resistant starch, and tannins (although high tannin content could be lowered by polishing grains up to three times). The frequency of polishing affected tannin and ash levels in all three sorghum varieties. Polishing frequency also affected the antioxidant capacity of polished sorghum grains. The findings from this study should be considered when determining appropriate applications for various sorghum-based food products.

## 1. Introduction

There is tremendous potential for developing sorghum as an alternative food ingredient due to the grain's adaptability, ease of cultivation, and the low costs associated with the latter. Several varieties of sorghum, each with its own distinctive physicochemical characteristics, are currently being cultivated in Indonesia. A previous study using six genotypes of sorghum indicated that the grains contained phenolic compounds and that depending on water stress or water scarcity during cultivation, the sorghum genotypes presented different responses [1]. According to Rao et al. [2], the content of dietary fibre, minerals, and starch in cookies made from 12 types of sorghum also varied. For example, cookies made from sorghum variety C43 had the highest starch and mineral (Mg, P, and K) contents, while those made from cultivars CSH 23, CSH 13R, and CSV 18R scored higher for overall acceptability and nutritional value. Similar findings were observed in flour and cookies made from two different varieties of bananas (i.e., showing different functional, physicochemical, and antioxidant properties) [3]. Nevertheless, research on the characteristics of different varieties of sorghum as they relate to food processing applications remains lacking.

Sorghum, with its high levels of dietary fibre, forms a key component of functional food and is recognised to have a range of health-promoting components. The protein content of this grain is equivalent to that of wheat (8–12%) and higher than that of rice (6–10%), while its fat content (2–6%) is higher than that of rice (0.5–1.5%). Yet, unlike other cereals, sorghum also has a testa (i.e., a dark (brown) grain coat that contains antinutritional compounds such as tannins) [4]. Tannins are polyphenolic chemicals that can form complexes with proteins and, in turn, lower protein quality and affect digestion [5]. Polyphenolic compounds can also inhibit the activity of digestive enzymes (especially amylase and trypsin) and thereby decrease starch digestibility. In addition to being antinutritional, tannins give rise to an astringent flavour in food products [6], which may explain the lower popularity of sorghum products among members of the public. Yet, tannin reduction efforts may improve nutritional quality (particularly the rate of starch and protein absorption) and the palatability or flavour of sorghum products.

Because of their role as a functional food, some tannin-containing sorghum genotypes have become consumer attractions [7]. It is, therefore, important to apply appropriate processing technologies to retain functional food components (e.g., antioxidants, minerals, fibres, oligosaccharides, and *β*-glucans (including nonstarch polysaccharide carbohydrates)) in ready-to-eat foods. During sorghum storage, pest or disease attacks may cause flavour deterioration and decrease the grain's nutritional content which, in turn, may render it unsuitable for consumption and thereby decrease its value [8]. Thus, to prolong the shelf life of sorghum grains, storage conditions should receive significant consideration.

Before being used to make food products, sorghum is generally required to undergo processing (either manually or mechanically) to remove its pericarp layer. This process, originally known as polishing (or pearling), forms part of dehulling and can affect product characteristics [9]. Polishing is an abrasive technique that gradually removes the grain coat (i.e., testa and pericarp) as well as aleurone, subaleurone, and germ layers to produce polished grains. Phytochemicals are mainly concentrated in the outer layers of the grain (i.e., the hull and bran) and are important lipid-soluble nutraceuticals. However, processing tends to decrease these contents as well [10]. For example, rice polishing affected the characteristics of the rice produced [11, 12], while sorghum polishing determined product quality (as nutritional value differed based on whether or not the sorghum had been milled) [13]. Moreover, the methodology used (i.e., mechanical vs. traditional processing) can also affect nutritional value. For example, both the polishing technique and the variety of sorghum used previously affected the nutritional value of crackers produced [14].

To date, regarding the properties of sorghum produced for various applications in Indonesia, there has been little research on the impact of sorghum cultivar or the extent of sorghum polishing. This study is thus aimed at researching the physical and chemical characteristics of sorghum in Indonesia (i.e., Bioguma, Numbu, and Super varieties) based on their polishing frequencies and seeing how different sorghum cultivars and milling processes affect the nutritional value of this grain. In obtaining a better understanding of the above, we could subsequently determine the best product applications for each sorghum variety based on their polishing frequencies.

## 2. Materials and Methods

### 2.1. Materials

Three varieties of sorghum grains (i.e., Bioguma, Numbu, and Super) were obtained from the Indonesian Sorghum Farmers Union (also known as Serikat Petani Sorgum Indonesia (SEPASI)) farmers in Tasikmalaya, West Java, Indonesia. The materials used for analysis included diphenyl picryl hydrazyl (DPPH), AlCl_3_, quercetin, Folin-Denis, Na_2_CO_3_, Na_2_SO_4_, CuSO_4_, TiO_2_, H_2_SO_4_, NaOH, Na_2_S_2_O_3_, H_3_BO_3_, BCG-MR indicator, HCl, tannin acid, phosphate buffer, *α*-amylase enzymes, *β*-amylase enzymes, acetone, methanol, ethanol, and aquades. The tools used included a UV-Vis spectrophotometer, analytical balance, Kjeldahl flask, Sokhlet, furnace, oven, vortex, micropipettes, centrifuge, polishing machine, grinder, and a sieve.

### 2.2. Manufacturing and Characterisation of Polished Sorghum

After harvesting, sorghum grains were sorted, weighed, and transferred into a polishing machine. The frequency was set according to specific treatment criteria. After polishing, the grain was cleaned, ground, and sieved before being analysed.

The current study followed an experimental method with a factorial randomised block design using sorghum variety (i.e., Bioguma (A1), Numbu (A2), or Super (A3)) as factor 1 and polishing frequency (i.e., once (B1), twice (B2), or three times (B3)) as factor 2. Observed variables included contents of ash, carbohydrates (by difference), dietary fibre, fat, moisture, resistant starch, and total proteins as well as antioxidant activity of DPPH, energy, and tannin levels.

### 2.3. Analyses

Moisture and ash contents were analysed (until reaching a constant weight [15]) by using a TGA-2000 thermogravimetric analyser (Navas Instruments, Conway, USA) at 105°C and 550°C, respectively. Protein content was measured via the Kjeldahl method, and a conversion factor of 6.25 was used to convert total nitrogen to percentage crude protein. Fat content was determined with an AOAC method (1990) and using the Soxhlet extraction technique (FOSS Soxtec™ extraction model, Sweden). Carbohydrate contents were calculated by difference. Specifically, the caloric value (kcal/100 g) of each loaf was calculated using Atwater's conversion factors (i.e., based on the caloric coefficients corresponding to protein (4 kcal/g), carbohydrate (4 kcal/g), and fat (9 kcal/g) contents) [16]. Dietary fibre analysis was performed using an enzymatic method (AOAC 1995), and total dietary fibre was calculated as the sum of insoluble and soluble dietary fibres.

Tannin content was calculated as the difference between total phenols and those remaining after precipitation with PVPP. The phenol content of the methanol extract solution was determined using the Folin-Ciocalteu method (according to Makkar (2003)). Tannic acid solutions in the range of 0.005–0.16 mg/ml were used to construct a calibration curve [17]. Quantification of resistant starch (RS) content was performed according to the method certified by AACC (2001) and AOAC (2002) and by using the RS assay kit (Megazyme International Ireland Ltd., Wicklow, Ireland). Antioxidant compounds were analysed according to the method of Thaipong et al. (2006). The DPPH assay was used to estimate the antioxidant capacity of phenolic compounds in the extracts.

## 3. Results and Discussion

### 3.1. Tannin Content

Sorghum typically contains tannins (i.e., phenolic compounds with high molecular weight (300–3000 Da)). Tannins are of interest to researchers due to their unique properties (e.g., binding to proteins [18, 19], inhibition of digestive enzymes or chelation, and reduced absorption of Fe and Zn ions [20]). Although these specific properties negatively affect digestibility and calorie intake, tannins also have beneficial properties relating to antioxidant activity (e.g., anticancer and anti-inflammatory effects) [1]. While intact, some varieties of sorghum contain high levels of tannins (up to 50 mg/g or more) that are concentrated in the pigmented testa. Based on the 1989 Codex regulations, the FAO and WHO suggested a safe limit of tannins at 0.5% (maximum) in sorghum grains and 0.3% (maximum) in sorghum flour [17].

**Figure 1:**
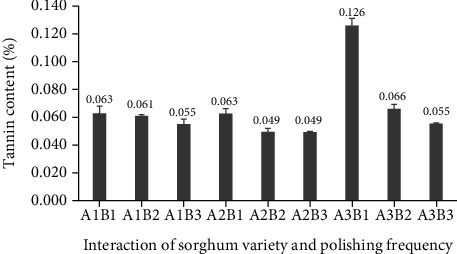
Tannin content of three varieties of sorghum grain processed with different polishing frequencies.

In the current study, the tannin content of polished sorghum grains differed according to both sorghum variety and frequency of polishing (Figure 1). The highest tannin content (0.126%) had been observed for once-polished Super cultivar grains, whereas the lowest tannin content (0.049%) had been observed for Numbu cultivar grains that had been polished two or three times.

**Figure 2:**
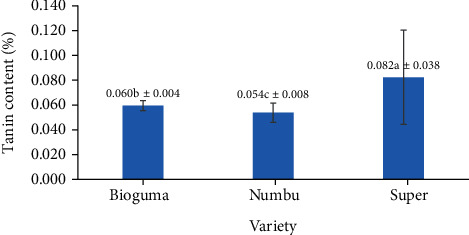
Tannin content of three varieties of polished sorghum grains.

When evaluated based on sorghum variety alone, the polished grains exhibited considerable variation in tannin levels (with Super and Numbu varieties showing the highest and lowest tannin levels, respectively) (Figure 2). This is due to variations in grain structure and size as well as other features. For example, 50 grains of 20 sorghum varieties may range between 1.1 and 1.9 g, indicating that grain size is influenced by the grain cultivar [17].

Similar to other cereals, sorghum grains contain phytic acid in the germ layer and tannins on the periphery [20]. Condensed tannins (proanthocyanidins) are especially found in sorghum varieties with a predominance of B1 and B2 genes (i.e., which have a pigmented testa) [17]. In the current study, only one variety (Super) had a pigmented testa. For the Numbu cultivar (whole sorghum), tannin concentration was previously found to be 0.91% [21]. Although some tannin-containing sorghum varieties may provide a bitter and astringent flavour [22], the bioactive components of these tannins can be attractive to functional food consumers [23]. It is known that tannin levels and grain structural properties depend on grain variety. For example, the Numbu variety is known to have a weight of 26.52 g/1000 grains and a skin thickness proportion of 0.8% [24]. Similarly, the Bioguma cultivar exhibits an almost identical grain size (with both Numbu and Bioguma cultivars being larger than the Super cultivar).

Grain size in plants also depends on genetic and environmental factors. For example, the distribution of pericarp, endosperm, and germ layers has been shown to differ depending on the cultivar and growing environment. On average, these layers, respectively, make up 8%, 82%, and 10% of sorghum grains. The pericarp is divided into three layers (i.e., epicarp, mesocarp, and endocarp). Then, just beneath the pericarp, a grain coat or testa layer is formed. Depending on the sorghum genotype, the thickness of the latter may vary (8–40 m) [25].

**Figure 3:**
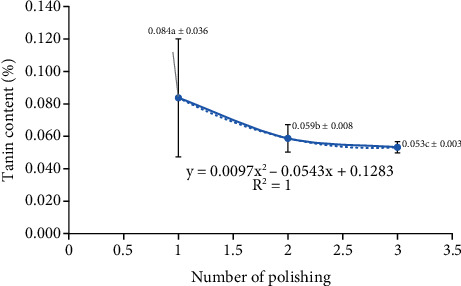
Tannin content of polished sorghum grains at different polishing frequencies.

It is this testa layer in sorghum grains that contain tannins. Before further processing, this layer can be removed by polishing (with abrasive and friction polishing representing the two most frequent methods). In the current study, a frictional polishing approach was used (i.e., generating friction between sorghum grains and causing erosion of bran layers from the grain surface). Tannin content was shown to be inversely proportional to the frequency of polishing (Figure 3). This was in agreement with a previous report indicating that decortication or removal of the grain husk layer significantly reduced tannin content by 79–92% [23].

The higher the polishing frequency, the cleaner the appearance of the grains and the lower the tannin content. This is because frictional intensity between sorghum grains increases with polishing frequency and becomes more optimal for testa removal. Three polishing rounds reduced tannin content to 0.053% (which was significantly lower than that of one or two polishing rounds). Dehulling is another important part of sorghum grain processing where ideally, all parts of the pericarp, testa, and germ should be removed with minimal damage to the endosperm. Pericarp thickness (in which endosperm hardness plays a role) is an important factor in both traditional and industrial-scale polishing processes. For example, a thick pericarp is easier to remove by grinding. Sorghum grains with a thick pericarp have certain disadvantages (e.g., sensitivity to mould and a tendency to damage during storage), while varieties with a thin pericarp are more tolerant to various types of damage (although the traditional polishing process takes twice as long for these varieties) [9].

### 3.2. Antioxidant Capacity (% Radical Scavenging Activity (RSA))

**Figure 4:**
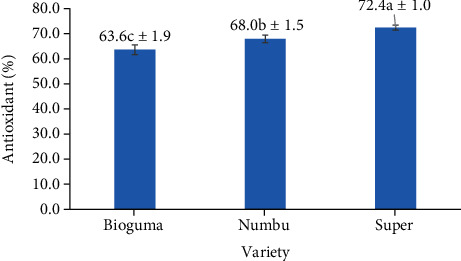
Antioxidant capacity (% RSA) of three varieties of polished sorghum.

There was no relationship between sorghum variety and frequency of polishing regarding antioxidant capacity as measured by the DPPH method. Furthermore, polishing frequency by itself did not affect the antioxidant capacity of polished sorghum grains. According to Dlamini et al. [23], decortication or removal of the outer skin of sorghum grains may significantly reduce antioxidants (including phenolic compounds and tannins) compared to that of whole grains. Unlike polishing frequency, sorghum variety heavily influenced the antioxidant capacity of polished grains. Specifically, the antioxidant capacity of the Super cultivar was higher than that of the Bioguma and Numbu cultivars (Figure 4). This Super cultivar also had a darker (reddish-brown) grain colour compared to the other two cultivars.

Antioxidant activity in sorghum grains is mainly associated with phenolic compounds, flavonoids, tannins, and anthocyanins [26]. Previously, total phenolics, flavonoids, and tannins in three types of white whole sorghum grains were shown to be 109.21–116.70 mg/100 g, 45.91–54.69 mg/100 g, and 1.39–21.79 mg/100 g, respectively [27], while black and brown sorghum grains had higher levels of antioxidants than blueberries, strawberries, and grapes (as measured by the oxygen radical absorption capacity (ORAC) method). Sorghum is therefore recognised as a potent scavenger of free radicals [28]. Moreover, sorghum varieties with a pigmented pericarp contain more phenolic compounds and have a higher antioxidant capacity than that of wheat. Phenolic compounds such as phenolic acid and flavonoids are abundant in sorghum (although flavonoids are unstable during the extrusion process) [26]. The majority of flavonoids in sorghum grains are concentrated in the outer section of the grain (pericarp). Flavonoid concentrations are thus determined by the thickness and presence of the testa which may, in turn, be influenced by genetic factors. For example, white grain sorghum genotypes have lower phenolic contents and simpler phenolic profiles compared to that of pigmented sorghum genotypes [1].

**Figure 5:**
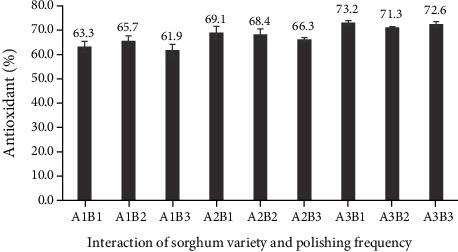
Antioxidant capacity of three polished sorghum varieties at different polishing frequencies.

As for other cereals, with chemical quantity dependent on variety, phenolic compounds are concentrated in the bran portion of sorghum grains [26]. Previous studies have also indicated that testa size is relatively large in brown sorghum genotypes SC319 and BR305, with correspondingly high levels of tannins [29]. In another study, the antioxidant activity of sorghum flour showed positive correlations with tocopherols, total vitamin E, total phenolic compounds, luteolinidine, apigeninidin, and total 3-deoxyanthocyanins (3-DXA) [30]. In the current study, antioxidant capacity values for the three polished sorghum varieties (with variations in polishing frequency) ranged from 61.9% to 73.2% (Figure 5). These high levels of phenolic antioxidants suggest that sorghum may be a good cereal choice for people with coeliac disease [25].

### 3.3. Dietary Fibre

**Figure 6:**
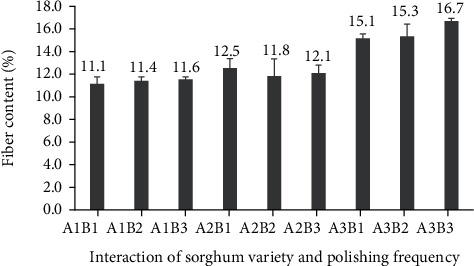
Dietary fibre content of three polished sorghum varieties at different polishing frequencies.

The current study, assessing polished sorghum grains, revealed no relationship between sorghum cultivar and polishing frequency for dietary fibre content (Figure 6). The highest dietary fibre content (16.7%) was detected in Super sorghum grains that were polished three times, while once-polished Bioguma grains had the lowest dietary fibre content (11.1%).

**Figure 7:**
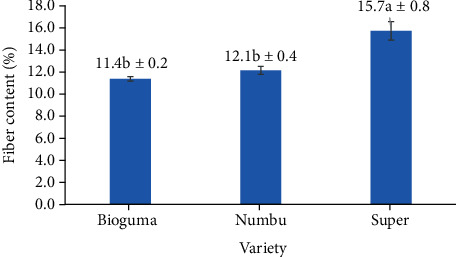
Dietary fibre content of three varieties of polished sorghum grains.

When assessing cultivars alone, the Super cultivar contained the highest fibre content (15.7%), followed by the Numbu (12.1%) and Bioguma (11.4%) varieties, respectively (Figure 7). Previous research showed that the content and composition of bioactive components, such as dietary fibre, varied widely among different cereals and cereal genotypes [31]. In addition to genotype (variety), the fibre content in cereals such as sorghum may be further influenced by the growing environment and processing conditions [32].

Unexpectedly, the polishing frequency did not affect dietary fibre levels in the current study (i.e., the level of dietary fibre in sorghum that was polished once was not significantly different from that of sorghum that was polished three times). As the nutritional components of dietary fibre in cereals are mostly found in the epidermis, the polishing process generally influences fibre contents. For example, the functional component of dietary fibre in Kawali sorghum flour has been shown to decrease according to the level of polishing [14].

### 3.4. Resistant Starch

**Figure 8:**
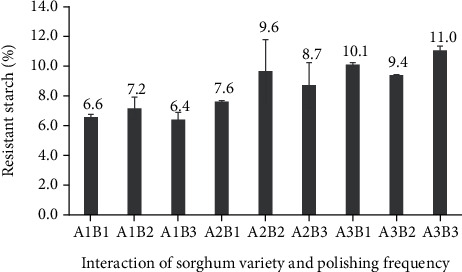
Resistant starch (%) in polished sorghum grains at different polishing frequencies.

Regarding the resistant starch content of polished sorghum grains, there had been no interaction between sorghum variety and polishing frequency (Figure 8). Resistant starch content had been highest (11%) for Super cultivar sorghum grains that were polished three times and lowest (6.4%) for similarly polished Bioguma grains. Resistant starch content is higher in sorghum than in wheat [33]. It can also be influenced by several factors including the ratio of amylose and amylopectin, pullulanase enzyme concentration, starch concentration, heating temperature, cycles of heating and cooling, storage conditions, and the presence of lipids or low molecular substances such as sugars (Sajilata et al.). In addition, processing techniques can affect the extent to which starch can be digested and absorbed and can, therefore, increase or decrease the content of resistant starch [34]. Because it has properties similar to soluble and insoluble fibres in the digestive tract, resistant starch levels in sorghum can play a health-related role by causing a slow release of glucose and reducing energy intake in intestinal cells [35].

**Figure 9:**
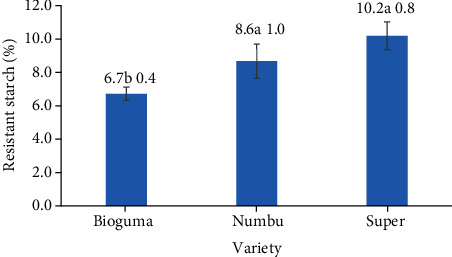
Resistant starch (%) of three varieties of polished sorghum grains.

However, the sorghum variety by itself had an overall impact on the amount of resistant starch in the grain (Figure 9). The resistant starch content of the Super cultivar (10.2%) was higher than that of Bioguma (6.7%) and Numbu (8.6%) cultivars. These findings were consistent with previous research showing varied resistant starch concentrations (0.31–65.65%) in 49 distinct sorghum types [19]. The resistant starch content of pigmented sorghum cultivars (such as Super) is known to be higher than that of white varieties. This is due to the interactions of sorghum tannins with starch that limit digestibility and increase resistant starch concentrations through the formation of insoluble complexes.

Although polishing frequency appeared to have no overall effect on resistant starch content, a slight tendency of increased resistant starch content with increased polishing frequency could be detected. These findings were in line with that of previous research which showed a positive correlation between the polishing level and starch content in cereals. Specifically, loss of the aleurone layer (containing fat and proteins) during the polishing process likely causes an increased concentration of starch in the endosperm [36].

### 3.5. Fat Content

**Figure 10:**
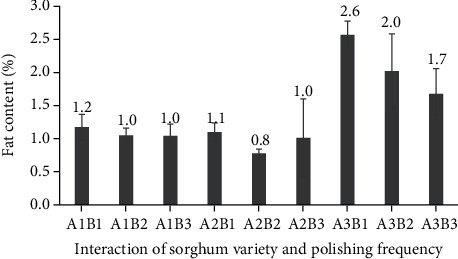
Fat content of three varieties of polished sorghum grains at different polishing frequencies.

The fat content of polished sorghum grains was mainly affected by cultivar and, to a lesser extent, polishing frequency (Figure 10). Depending on whether it was polished once, twice, or three times, sorghum fat content ranged from 0.8% to 2.6%. The fat content of whole sorghum grains (mainly concentrated in the scutella portion of the germ [23]) has been shown to range from 1.0% to 3.4% [28]. The fat content of sorghum is known to be higher compared to that of other grains. This content can be distributed in varying amounts within different parts of the kernel (e.g., the majority of the fat is found in the germ (76.2%), followed by the endosperm (13.2%) and the pericarp (10.6%)) [29].

**Figure 11:**
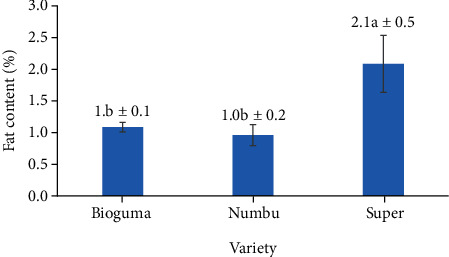
Fat content of three varieties of polished sorghum grains.

The fat content of polished sorghum varied depending on the cultivar (Figure 11). At 1%, the fat content of polished Bioguma and Numbu grains had been considerably lower than the 2.1% fat content of polished Super grains. The grain size of the latter had also been smaller compared to that of Bioguma and Numbu varieties. Yet, despite this smaller size, Super cultivar grains were expected to have a larger germ (where the majority of lipids would be concentrated). The chemical composition of sorghum grains is influenced by physical, genetic, and environmental characteristics. In the current study, reddish-coloured grains (Super variety) had a higher fat content compared to white varieties (Bioguma and Numbu). This differed from the findings of Bernardo et al., who showed that sorghum varieties with a white pericarp had the highest lipid contents. Sorghum lipids, particularly phytosterols and policosanols, can act as bioactive molecules, which are advantageous to health [29].

Although the lipid content of polished sorghum was largely unaffected by the frequency of polishing, an unsubstantial difference could be detected in that fat content slightly decreased with each extra polishing. The polishing procedure performed in this study also removed some pericarp and germ layers (i.e., resulting in lower fat content compared to that of whole sorghum). It is known that fat levels in cereals may affect grain quality during storage and that, in general, lower fat content supports a longer cereal shelf life.

Germination rate, free fatty acid concentration, and MDA have all been positively associated with grain storage. During grain storage, various metabolic processes may cause the build-up of reactive oxygen species (ROS) which, in turn, can target membrane lipids and cause alterations in nutritional reserves, lipid contents, acidity, and lipoxygenase activity (among other degenerative processes). Free radical-mediated lipid peroxidation is usually the principal source of cell membrane damage during storage.

### 3.6. Protein Content

**Figure 12:**
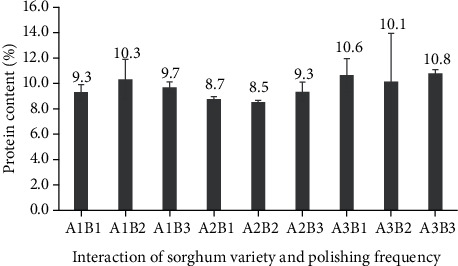
Protein content of three varieties of polished sorghum grains at different polishing frequencies.

The protein content of polished sorghum (ranging between 8.5% and 10.8%) was not affected by sorghum variety or the frequency of polishing (Figure 12). Whole sorghum has previously been shown to contain 12% protein [29], and it generally has similar protein contents to other cereals. However, the removal of certain grains during polishing (especially in the pericarp) can decrease protein content in polished sorghum grains.

**Figure 13:**
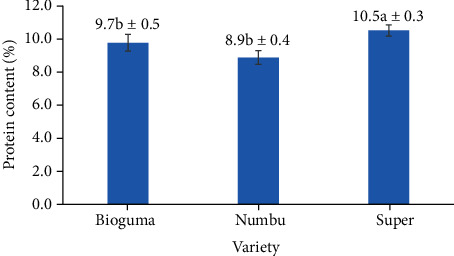
Protein content of three varieties of polished sorghum grains.

The protein content of polished sorghum was influenced by the sorghum cultivar in different ways (Figure 13). Protein content was higher in the Super cultivar (10.5%) than in either Bioguma (9.7%) or Numbu (8.9%) cultivars (both of which were white cultivars). These findings were in line with previous research which found variation in protein content of six distinct sorghum cultivars [32]. When tannins and proteins coexist, protein bindings can occur that consequently lower protein digestibility *in vivo*. Thus, as tannin concentration increases, *in vivo* protein digestibility decreases (as proteins bound to tannins are less easily broken down by protease enzymes) [28].

The protein content of polished sorghum grains was not affected by polishing frequency. According to Ratnavathi and Komala, most of the protein in sorghum grains can be found in the endosperm. The latter, consisting of four layers (i.e., aleurone, peripheral endosperm, vitreous endosperm (hard), and floury endosperm (soft)), is unaffected by polishing. The aleurone layer is one of the layers beneath the grain coat, and it is rich in protein, enzymes, lipids, B-complex vitamins, and minerals. The nutritional quality of protein in grains is influenced by processing methods, while protein digestibility and bioavailability can be improved by milling, fermentation, and germination [37].

### 3.7. Ash Content

**Figure 14:**
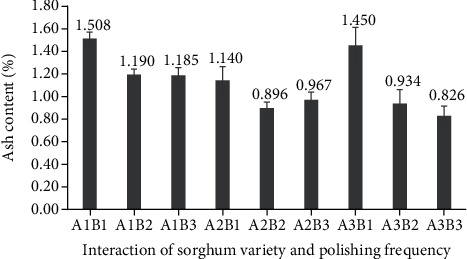
Ash content of three varieties of polished sorghum grains at different polishing frequencies.

Relationships between ash content, sorghum variety, and polishing frequency could be detected in the current study (Figure 14). The highest ash content was found in once-polished Bioguma (1.45%) and Numbu (1.50%) cultivars, while the lowest ash content was found in twice-polished Numbu (0.89%) and thrice-polished Super (0.82%) cultivars. Ash content refers to the mineral content of a food item. Mineral deficits in the diet can affect mental and physical development, work performance, and infectious illness (particularly in young, pregnant, and breastfeeding women).

**Figure 15:**
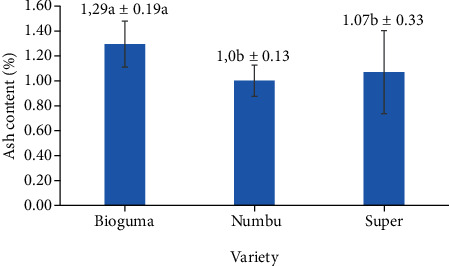
Ash content of three varieties of polished sorghum.

The ash content of polished sorghum varied according to cultivar (Figure 15). The ash level was higher in the Bioguma cultivar (1.29%) than in either Numbu (1.0%) or Super (1.07%) cultivars. Nonetheless, these ash levels were lower than that of previous studies which reported 1.49% and 1.35% for Bioguma and Numbu cultivars, respectively. Cultivar-specific variables, environmental conditions, and the interplay between genotype and the environment have all been shown to influence ash content. Depending on the sorghum cultivar, mineral amounts can vary: Cu (0.33–1.01 mg/kg), Fe (4.7–14.9 mg/kg), K (3.4–6.9 g/kg), Mg (0.79–1.47 g/kg), Ca (0.06–0.19 g/kg), P (1.79–2.78 g/kg), and S (0.67–1.01 g/kg) [37, 38]. Sorghum is thus a good source of minerals, with potassium, phosphorus, magnesium, and iron being the most prevalent in this grain [39].

**Figure 16:**
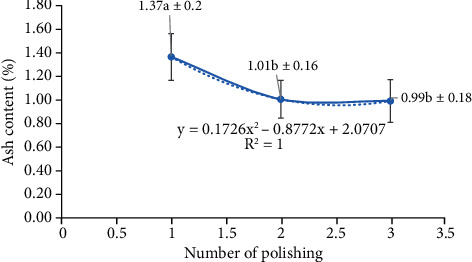
Ash content of polished sorghum grains at various polishing frequencies.

The amount of ash in polished sorghum was also affected by the frequency of polishing (i.e., an increased frequency reduced ash content) (Figure 16). This finding was in line with previous research reporting higher levels of ash in whole sorghum flour (1.88%) than in polished sorghum flour (0.68%). The ash content of sorghum thus reflects the mineral richness of this grain, and the polishing process, in turn, has a large impact on this mineral content. This is because minerals are largely contained in the bran and embryo (which are lost during the polishing process), and a higher polishing frequency, therefore, results in lower mineral content. The bran and germ regions of grains also contain the majority of vitamins. During overmilling and high levels of extraction commonly observed in the manufacturing of white bread flour, both the aleurone and germ layers (which are rich in vitamins, minerals, and other phytochemical elements) are eliminated. However, in terms of overall nutritional quality, it should be noted that the amount of minerals absorbed is not always proportional to the amount of minerals consumed (as absorption is governed by availability). Although polishing reduces micronutrient content, it also makes it easier for the body to absorb such components. Furthermore, enrichment or fortification processes may be implemented to compensate for micronutrient deficits produced by the milling process.

### 3.8. Moisture Content

**Figure 17:**
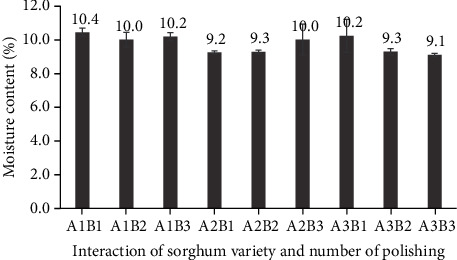
Water content (by difference) of three varieties of polished sorghum grains at different polishing frequencies.

The moisture content of polished sorghum grains was unaffected by either cultivar or polishing frequency. Specifically, the moisture content was found to be between 9.1% and 10.4% (Figure 17). Previous research has shown that the moisture content of sorghum flour varies in the range of 10.1–14.4%, with an average value of 11.5% [17]. All values obtained in the current study had been below 15%, which is the upper limit established for sorghum flour.

### 3.9. Carbohydrate Content

**Figure 18:**
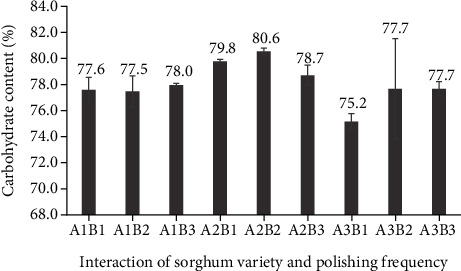
Carbohydrate content (by difference) of three varieties of polished sorghum grains at different polishing frequencies.

In the current study, carbohydrate content was calculated by difference. The carbohydrate content of polished sorghum grains (75.2–80.6%) was not affected by the frequency of polishing (Figure 18).

**Figure 19:**
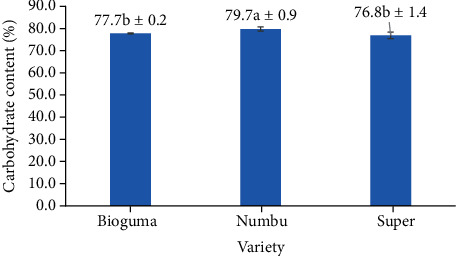
Carbohydrate content (by difference) of three varieties of polished sorghum.

In contrast, the carbohydrate content of polished sorghum varied according to cultivar (Figure 19). The total carbohydrate content of whole sorghum grain has been reported as 74.63 g/100 g, with a total sugar content of 3.39 g/100 g, 3–4% cellulose content, and 55–75% starch content [29]. While sorghum contains just 3.4–7.3% of noncellulosic polysaccharides ((1,3;1,4)-b-glucans and arabinoxylans), these compounds make up more than 10% of other cereals such as wheat, barley, and oats. As shown in Figure 19, while there was no statistical difference between carbohydrate content levels for Bioguma and Super cultivars, their values were significantly lower than that of the Numbu cultivar. The size, composition, and structure of grains can all be affected by cultivar.

Carbohydrates are largely concentrated in the endosperm (although with different amounts and types according to species). For example, xyloglucan can be detected in the testa and aleurone of some cultivars but not in the endosperm. Variable levels of *β*-glucan, arabinose, xylose, galactose, galacturonic acid, starch, and free sugar have been detected in three varieties of sorghum. It was concluded that two major factors contributed to the total carbohydrates, namely, the cultivar (74%) and the environment (14%).

The frequency of polishing did not influence carbohydrate content as most of the carbohydrates in cereal grains are found in the endosperm (which is left intact after polishing). Contrastingly, the removed exterior parts of the grain (i.e., pericarp, testa, and aleurone) contain very few carbohydrates.

Starch serves as the main energy source in cereal grains. However, processing methods can affect both the digestibility and calorific value of starch. For example, when ground into flour, the total energy content in wheat is increased. This is because grinding increases the digestibility of carbohydrates by removing type 1 resistant starch trapped in the outer layer of grains. In addition, phytochemical compounds in the germ and epidermis may inhibit the activity of amylase and protease enzymes (i.e., reducing the total available calories) [34]. Finally, cultural preferences for processing different grain varieties can also affect their carbohydrate availability and glycaemic indices [34].

When compared to other sorghum varieties, the Super cultivar was found to have the highest protein content. Furthermore, its antioxidants (including tannins), nutritional fibre, and starch resistance had also been higher. Excessive quantities of tannins in food may, however, cause astringent flavours. To avoid this, tannin content can be reduced by polishing the grains up to three times. Subsequent evaluation of flour quality (as produced by each cultivar) and determination of potential appropriate applications for different cultivars represent an important next step in this research.

## 4. Conclusions

The sorghum Super cultivar was found to contain the highest levels of tannins, proteins, antioxidants, dietary fibres, resistant starch, and fat contents. High tannin content could be lowered by polishing grains up to three times. These findings may aid considerations regarding the best applications for various sorghum-based food items.

## Data Availability

The data used to support the findings of this study are available from the corresponding author upon request.
